# Two new species and one newly recorded species of *Uloma* Dejean, 1821 from Zhejiang, China (Coleoptera, Tenebrionidae, Ulomini)

**DOI:** 10.3897/zookeys.607.7836

**Published:** 2016-07-28

**Authors:** Shanshan Liu, Guodong Ren

**Affiliations:** 1 The Key Laboratory of Zoological Systematics and Application, College of Life Sciences, Hebei University, 071002, Baoding, Hebei, P. R. China

**Keywords:** China, new species, taxonomy, Tenebrionidae, *Uloma*

## Abstract

Two new species of the genus *Uloma* Dejean, 1821, *Uloma fengyangensis* **sp. n.** and *Uloma acrodonta* **sp. n.**, are described and figured from Zhejiang Province of China. *Uloma bonzica* Marseul, 1876 is recorded from China for the first time. A key to the known *Uloma* species from Zhejiang of China and a list of *Uloma* species from China are provided.

## Introduction

The tenebrionid genus *Uloma* was established by Dejean (1821) based on *Uloma culinaris* (Linnaeus, 1758) from Germany. *Uloma* includes more than 200 described species that are widely distributed in nearly all zoogeographical regions of the Old and New World and is particularly speciose in the tropics (Schawaller 2015). There are 34 species of the genus recorded in China presently. They were described by Wiedemann (1821), Hope (1831), Fairmaire (1882), Gebien (1914), Kaszab (1941a, 1941b, 1954, 1980), Nakane (1968), Masumoto (1982), Masumoto and Nishikawa (1986), Ren and Liu (2004), Ren and Yin (2004), Liu and Ren (2007, 2008, 2013) and Liu et al. (2007).

Two new species of *Uloma*, *Uloma fengyangensis* sp. n. and *Uloma acrodonta* sp. n., were collected from Mt. Fengyangshan in Zhejiang Province of China. *Uloma bonzica* Marseul, 1876, a species newly recorded from China, was also sampled at the same locality. The two new species are described and figured in this paper, and a dorsal habitus of the new record is also presented. In order to help with the identification of the *Uloma* species from Zhejiang, a key to its species known from Zhejiang Province is provided.

## Materials and methods

The terminology of morphological structures follows that of Schawaller (1996) and Matthews and Bouchard (2008). The photographs were taken with a Leica M205A stereomicroscope equipped with a Leica DFC 450 digital microscope camera. All measurements were made in millimetres. The types and other examined specimens are deposited in the Museum of Hebei University (Baoding, China; MHBU), the Muséum National d’Histoire Naturelle (Paris, France; MNHN) and the National Museum of Nature and Science (now in the Masumoto Collection, Tokyo, Japan; NMNS).

## Taxonomy

### Uloma fengyangensis sp. n.

Taxon classificationAnimaliaColeopteraTenebrionidae

http://zoobank.org/8251A721-A6B7-43A7-AB48-8461A4477DE4

Figs 1A , 2

#### Type material.

Holotype, ♂ (MHBU), labelled “25 July 2007; China, Zhejiang, Longquan County, Mt. Fengyangshan; H. Y. Liu and Z. H. Gao lgt.; the Museum of Hebei University” (white, rectangular, printed, in Chinese); “Holotype; *Uloma fengyangensis* sp. n. Liu & Ren det. 2015” (red, rectangular, printed and handwritten).

#### Diagnosis.

The new species is characterized by the following: mentum broadly cordate, with several short medial hairs and a pair of semi-circular hairy patches on near lateral margins; antennomere 5–10 sublinearly truncate, with one long groove on each inner side; pronotum with a pair of low protuberances on lateral margins and posterior margin of anterior impression respectively; metatarsomere 1 significantly longer than 4; apicale of aedeagus with a shallow depression on centre at basel 1/3, parallel–sided at apical 2/3 in dorsal view.

#### Description.

**Figure 1.**
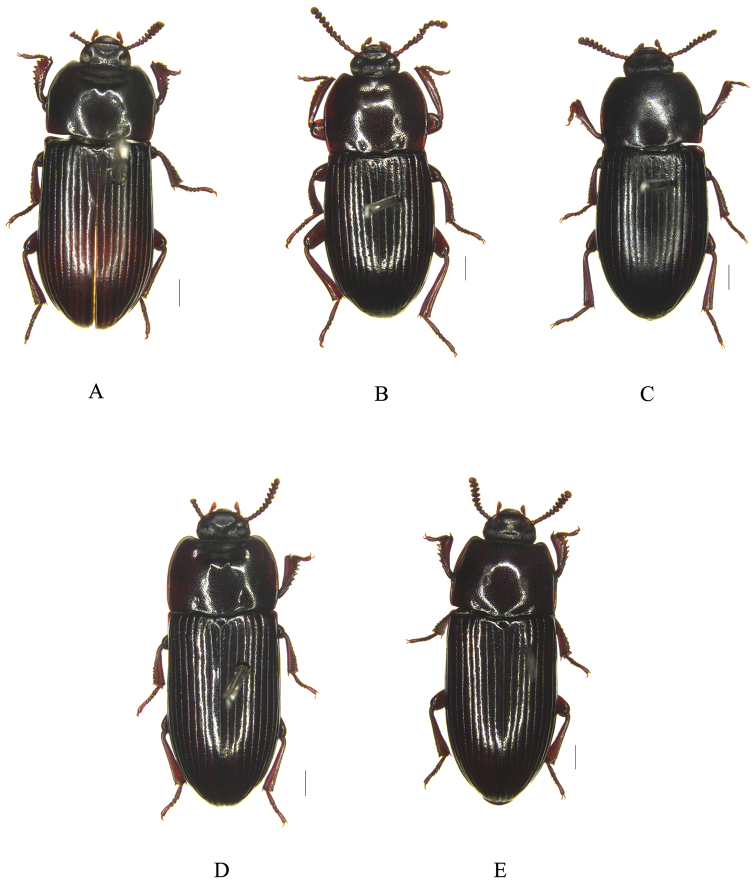
Habitus, dorsal view. **A** *Uloma fengyangensis* sp. n., male **B** *Uloma acrodonta* sp. n., male **C** *Uloma acrodonta* sp. n., female **D** *Uloma bonzica* Marseul, 1876, male **E** *Uloma bonzica* Marseul, 1876, female. Scale bars 1 mm.

**Figure 2.**
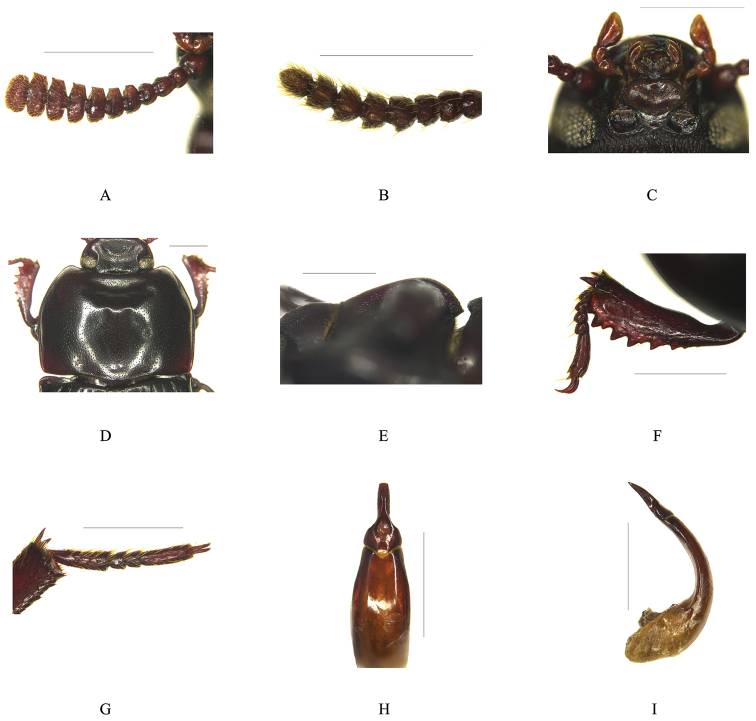
*Uloma fengyangensis* sp. n., male. **A** Antenna, ventral view **B** Antennomere 5 to 10, lateral view **C** Ligula and mentum, ventral view **D** Pronotum, dorsal view **E** Posternal process, lateral view **F** Protibia, dorsal view **G** Metatarsus, dorsal view **H** Apicale of aedeagus, dorsal view **I** Aedeagus, lateral view. Scale bars 1 mm.

Male (Fig. 1A). *Head* transverse, with small punctures in apical half, and with sparse large punctures in basal half. Labrum trapezoidal, sparsely punctate, scattered with long and yellow hairs. Clypeus densely and distinctly punctate, anterior margin weakly emarginate, slightly elevated with two small ridges. Frontoclypeal suture deeply impressed. Genae slightly convex and extended, temples reduced. Eyes transverse, with at least 3–4 facets at narrowest point in lateral view; distance between eyes approximately 2.7 times longer than their diameter. Frons weakly convex but depressed on centre, with large punctures. Mentum (Fig. 2C) broad cordate, weakly emarginate at anterior margin, slightly concave with several short medial hairs, with a pair of semi-circular hairy patches on external sides. Ligula (Fig. 2C) deeply emarginate anteriorly, depressed in the middle with sparse long hairs. Terminal maxillary palpomere somewhat knife-shaped. Antennae (Fig. 2A) long, reaching to the middle of pronotum; antennomere 1 thick, 2 very short, 3 long and narrow, 4 short, 5 - 10 gradually widening, forming a more or less distinct club, 8–10 extremely transverse, nearly rectangular, 11 transverse-oval; 5–10 sublinearly truncate with one long groove at each innerside (Fig. 2B); ratio of the length (and the width) of antennomeres 2–11 as follows (mm): 8 (10): 9 (10): 7 (12): 7.5 (15): 7.5 (16): 8 (18): 8 (20): 9 (22): 9 (21): 12 (19).

*Pronotum* (Fig. 2D) transverse, nearly 1.5 times as wide as long, widest at middle, with small punctures widely spaced on centre and becoming denser toward sides, with reticulate microsculptures. Pronotum with a small and deep anterior impression and a pair of low protuberances on both sides and posterior margin of impression respectively, and with a shallow groove in the middle of posterior margin. Anterior margin emarginate with narrow border only at both apices, without border in the middle 1/3, and with dense short hair fringes. Lateral margins arcuate, strongly narrowing forward and less so from widest point to base, with narrow border. Basal margin slightly convex, with a pair of oblique shallow impressions. Anterior angles subrectangular, posterior angles obtuse. Prosternum with dense large and partly confluent punctures, posternal process (Fig. 2E) rounded in lateral view, smoothly descended at apex, with sparse small punctures centrally. Propleuron with long wrinkles and large confluent punctures.

*Scutellum* subtriangular, with very sparse small punctures. Elytra nearly parallel–sided; elytra distinctly punctato–striate, punctures of elytral rows small and only somewhat broader than stripes; intervals slightly convex, distinctly and sparsely punctate with several transverse wrinkles, lateral margins visible only at humeri in dorsal view. Hind wings developed.

*Protibia* (Fig. 2F) with two equal apical spurs; slightly concave, narrow at base, then strongly and gradually explanate on both inner and outer edges; inner edge weakly emarginate at base, slightly protruding to inner apex, fringed with yellow short hairs becoming denser and longer toward apex; outer edge with 8–9 sharp denticulations at apical 3/4 scattered with short hairs; dorsal surface with a long depression near apex and large sparse and confluent punctures; ventral surface with a row of several small sharp protuberances and short sparse hairs. Mesotibia feebly and gradually expanding toward apex, outer edge with small denticles and sparse long hairs. Metatibia feebly and gradually expanding toward apex, outer edge with sparse long hairs. Length ratios of metatarsomeres (Fig. 2G) 1 to 4 as follows: 35: 9: 9: 28.

*Abdominal ventrites* finely densely punctate, punctuation larger and subcontiguous towards lateral portions.

*Aedeagus* (Fig. 2H–I) with basale subparallel–sided; apicale slender, gradually narrowing with a shallow depression on centre at basel 1/3, parallel–sided at apical 2/3, truncate at apex in dorsal view, with a longitudinal depression on centre in ventral view, slightly curved in lateral view.

Female. Unknown.

Body length: 11.0 mm; elytral width at widest point: 4.5 mm.

#### Etymology.

The species epithet refers to the Mt. Fengyangshan where the species was collected.

#### Remarks.

The new species is similar to *Uloma reticulata* Liu, Ren & Wang, 2007, but can be distinguished from the latter by the following characters: (1) male mentum broadly cordate, slightly concave with several short medial hairs, with a pair of semi-circular hairy patches on near lateral margins in the new species (subhexagonal, with cordate convex in middle, without hairy patch in *Uloma reticulata*); (2) male antennomere 5–10 sublinearly truncate, with one long groove on each inner side in the new species (5–9 sublinearly truncate with one long groove in *Uloma reticulata*); (3) male pronotum with a pair of low protuberances on lateral margins and posterior margin of anterior impression respectively, anterior angles subrectangular in the new species (anterior impression of pronotum without protuberance in *Uloma reticulata*); (4) male metatarsomere 1 significantly longer than 4 in the new species (1 almost as long as 4 in *Uloma reticulata*); (5) apicale of aedeagus gradually narrowing with a shallow depression on centre at basel 1/3, parallel–sided at apical 2/3 in dorsal view in the new species (gradually narrowing from base to apex, then slightly widening nearly apex in *Uloma reticulata*).

### Uloma acrodonta sp. n.

Taxon classificationAnimaliaColeopteraTenebrionidae

http://zoobank.org/BA19A5DB-F014-4EAF-A958-33C507E5491A

Figs 1B–C , 3

#### Type material.

Holotype, ♂, labelled “19 July 2012; China, Zhejiang, Longquan County, Mt. Fengyangshan; X. Wang and J. Jiao lgt.; the Museum of Hebei University” (white, rectangular, printed, in Chinese). Paratypes, 1♂1♀, labelled as holotype. All types have additional label “Holotype (and Paratype, respectively), *Uloma acrodonta* sp. n. Liu & Ren det. 2015” [red (and Paratype with yellow, respectively), rectangular, printed and handwritten], and all of them are deposited in MHBU.

#### Diagnosis.

The new species is characterized by the following: clypeus slightly elevated with two small ridges; antennomeres 5 and 7 obviously sharply protruding at inner border; pronotum with a small and shallow anterior impression; protibia broader, with 8–9 sharp large denticulations at apical 2/3 of outer edge; last ventrite with a shallow impression.

#### Description.

**Figure 3.**
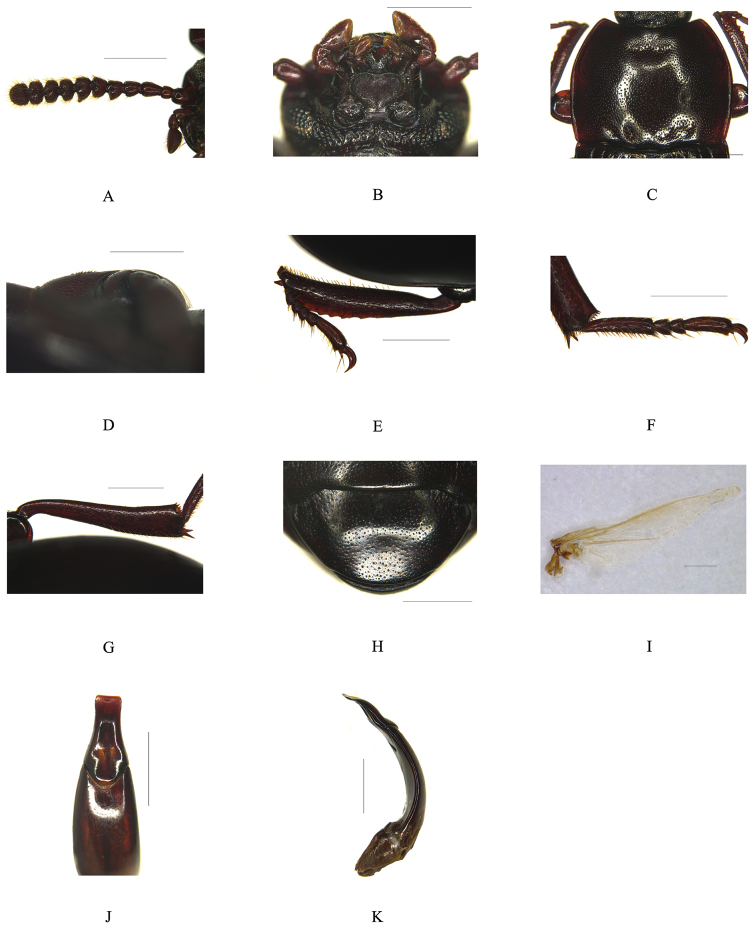
*Uloma acrodonta* sp. n., male. **A** Antenna, ventral view **B** Ligula and mentum, ventral view **C** Pronotum, dorsal view **D** Posternal process, lateral view **E** Protibia, dorsal view **F** Metatarsus, dorsal view **G** Metatibia, dorsal view **H** Last ventrite, ventral view **I** Hind wing, dorsal view **J** Apicale of aedeagus, dorsal view **K** Aedeagus, lateral view. Scale bars 1 mm.

Male (Fig. 1B). *Head* nearly hexagonal, with dense small punctures in apical half, and with dense large punctures in basal half. Labrum trapezoidal, sparsely punctate, scattered with long yellow hairs. Clypeus densely and finely punctate, anterior margin weakly emarginate, slightly elevated with two small ridges. Frontoclypeal suture deeply impressed. Genae slightly convex and extended, temples reduced. Eyes transverse, with at least 2–3 facets at narrowest point in lateral view; distance between eyes approximately 3.5 times longer than their diameter. Frons convex but slightly depressed on centre, with large coarse punctures. Mentum (Fig. 3B) cordate, truncate basally, weakly emarginate at anterior margin, flat with fine transverse wrinkles in middle, with a pair of crescent-shaped hairy patches on external sides. Ligula (Fig. 3B) deeply emarginate anteriorly, depressed in middle with sparse long hairs. Terminal maxillary palpomere knife-shaped. Antennae (Fig. 3A) long, reaching to the middle of pronotum; antennomere 1 thick, 2 very short and subquadrate, 3 slender, 4 short, 5–10 gradually widening, forming a more or less distinct club, 11 semi-spherical; 5, 7 obviously sharply protruding at inner border; ratio of the length (and the width) of antennomeres 2–11 as follows: 8.5 (9): 18 (12.5): 12 (12.5): 11 (16.5): 11 (16): 11 (20.5): 11 (18): 10.5 (18): 10.5 (18): 15 (17.5).

*Pronotum* (Fig. 3C) slightly transverse, subquadrate, nearly 1.2 times as wide as long, widest at middle, with sparse small punctures widely spaced on centre and becoming denser toward sides. Pronotum with a small and extremely shallow anterior impression without protuberances. Anterior margin emarginate with narrow border only at both apices, without border in the middle 1/3, and with dense short hair fringes. Lateral margins arcuate, strongly narrowing forward and less so from widest point to base, with broad border. Basal margin slightly convex. Anterior angles sharp, posterior angles rectangular. Prosternum with sparse and large punctures, posternal process (Fig. 3D) rounded in lateral view, smoothly descended at apex, with coarse transverse wrinkles and two rows of short yellow hairs on centre. Propleuron with long wrinkles and large punctures. Metasternum very short.

*Scutellum* subtriangular, with sparse and small punctures. Elytra nearly parallel–sided; elytra distinctly punctato–striate, punctures of elytral rows small and only somewhat broader than stripes; intervals slightly convex, distinctly and sparsely punctate with several transverse wrinkles, lateral margins visible only at humeri in dorsal view. Hind wing (Fig. 3I) vestigial, narrow, and short.

*Protibia* (Fig. 3E) with two equal apical spurs; nearly straight, narrow at base, then feebly and gradually explanate on both inner and outer edges; inner edge weakly emarginate at base, distinctly protruding to inner apex, fringed with yellow short hairs becoming denser and longer toward apex; outer edge with 8–9 sharp denticulations at apical 2/3 scattered with short hairs; dorsal surface with a long depression near apex and large sparse and not confluent punctures; ventral surface with a row of several small sharp protuberances and short sparse hairs. Mesotibia feebly and gradually expanding toward apex, outer edge with small denticles and sparse short hairs. Metatibia (Fig. 3G) slightly curved, feebly and gradually expanding toward apex, outer edge smooth without denticles and hairs. Length ratios of metatarsomeres (Fig. 3F) 1 to 4 as follows: 46: 10: 9.5: 32.

*Abdominal ventrites* finely and densely punctate, punctuation larger and subcontiguous towards lateral portions; last ventrite (Fig. 3H) with a very shallow impression.

*Aedeagus* (Fig. 3J–K) with basale parallel–sided; apicale broad at base, then gradually feebly narrowing towards apex, subparallel–sided near apical, truncate and semi-circularly depressed at apex in dorsal view, with a longitudinal depression on centre in ventral view, slightly curved in lateral view.

Female (Fig. 1C). Mentum subcordate, with V-shaped convex on centre, without hair. Clypeus without ridges. Antennomere not protruding to inner border. Pronotum without anterior impression. Protibia with shape similar to that of male, inner edge not protruding to inner apex. Metatibia straight. Last ventrite without impression.

Body length: 12.5–13.0 mm; elytral width at widest point: 4.5 mm.

#### Etymology.

The species epithet refers to the sharply protruding at inner border of antennomere 5 and 7.

#### Remarks.

The new species is most similar to *Uloma quadratithoraca* Liu & Ren, 2008, but can be distinguished from the latter by the following characters: (1) male clypeus slightly elevated with two small ridges in the new species (without ridge in *Uloma quadratithoraca*); (2) male antennae long, reaching to the middle of pronotum, antennomeres 5 and 7 obviously sharply protruding at inner border in the new species (5 and 7 not protruding in *Uloma quadratithoraca*); (3) male pronotum with a small and shallow anterior impression in the new species (without anterior impression in *Uloma quadratithoraca*); (4) male protibia distinctly broader, with 8–9 sharp large denticulations at apical 2/3 of outer edge in the new species (narrower, with 8–9 undulant denticulations at apical 1/2 in *Uloma quadratithoraca*); (5) male last ventrite with a shallow impression in the new species (without impression in *Uloma quadratithoraca*).

Moreover, five additional species (*Uloma intriconicula* Liu, Ren & Wang, 2007, *Uloma metogana* Ren & Yin, 2004, *Uloma takagii* Masumoto & Nishikawa, 1986, *Uloma rubripes rubripes* (Hope, 1831) and *Uloma rubripes minor* Gebien, 1914) are known to occur in China and its surrounding areas with antennomere 5 and 7 obviously sharply protruding at inner border. The new species is easily distinguished from them based on shape differences in the male pronotum, pronotal anterior impression, protibia, metatibia, ridges of clypeus, and whether or not the pronotal anterior impression exists in female.

### Uloma bonzica

Taxon classificationAnimaliaColeopteraTenebrionidae

Marseul, 1876

Fig. 1D–E

Uloma bonzica Marseul, 1876: 114; Nakane 1956: 36; Masumoto and Nishikawa 1986: 24; Löbl et al. 2008: 302.
Uloma bonzica robustior Nakane, 1956: 167.

#### Material examined.

Types, 1♂1♀ (MNHN, the Marseul Collection), Nzgzzalli. Others: 1♂1♀ (MHBU), China, Zhejiang, Longquan County, Mt. Fengyangshan, 25 July 2007, H. Y. Liu and Z. H. Gao lgt.; 2♂♂1♀ (NMNS), Fujitani Iga-Ueno, Mie, 3 November 1984, K. Ishida lgt.

#### Description.

Male (Fig. 1D). Mentum subhexagonal, slightly emarginate at anterior margin, with V-shaped convex and fine transverse wrinkles in the middle, without hair. Ligula depressed in the middle with dense hairs and hairy area wide. Antennae reaching to basal 1/3 of pronotum; antennomere 11 nearly semi-spherical. Pronotum nearly 1.5 times as wide as long, widest at basal 1/3; pronotum with a small deep anterior impression and a pair of low protuberances on both sides and posterior margin of impression respectively. Protibia with two unequal apical spurs, inner edge nearly straight at base, distinctly protruding to inner apex; outer edge with 7–8 sharp denticulations at apical 2/3; dorsal surface with large, dense and confluent punctures. Female (Fig. 1E) ligula with dense hairs and hairy area wide, pronotum without anterior impression, last ventrite without apical groove.

#### Remarks.

The Chinese specimens almost conform to the original description by Marseul (1876), but body length (11.7 mm) is slightly longer than that of the specimens from Japan (10.6 mm), and also the ratio of the distance between the eyes versus their diameter (*ca.* 2.5) less than that of the specimens from Japan (*ca.* 2.8). However, we think these two characters as intraspecific differences.

#### Distribution.

China: Zhejiang (new record); Japan (Marseul 1876, Gebien 1940, Nakane 1956, Masumoto and Nishikawa 1986, Kwon and Choi 1986, Löbl et al. 2008); Korea (Masumoto and Nishikawa 1986, Kwon and Choi 1986, Löbl et al. 2008).

##### Key to known species of the genus *Uloma* from Zhejiang Province of China

**Table:** 

1	Male protarsomere 3 with a lobed protuberance (fig. 6b, in Gebien 1914). China (Zhejiang, Guangxi, Taiwan), Himalayas, Oriental Region, Afrotropical Region	***Uloma polita* (Wiedemann, 1821)**
–	Male protarsus normal, protarsomere 3 without protuberance	**2**
2	Male ligula with dense hairs and hairy area wide (fig. 25, in Masumoto and Nishikawa 1986)	**3**
–	Male ligula with several sparse long hairs (Fig. 2C)	**4**
3	Inner edge of male protibia strongly and rather abruptly emarginate at base; last ventrite of female with a deep apical groove (figs 34–35, in Masumoto and Nishikawa 1986). China (Zhejiang, Guangxi, Hainan, Fujian, Taiwan), Oriental Region	***Uloma excisa excisa* Gebien, 1914**
–	Inner edge of male protibia nearly straight at base; last ventrite of female without apical groove (fig. 24, in Masumoto and Nishikawa 1986). China (Zhejiang), North Korea, South Korea, Japan	***Uloma bonzica* Marseul, 1876**
4	Male antennomere 5 and 7 obviously sharply protruding at inner border; metasternum very short; hind wings vestigial, narrow and short (Fig. 3A, I). China (Zhejiang)	***Uloma acrodonta* sp. n.**
–	Male antennomere 5 and 7 not protruding at inner border	**5**
5	Male mentum without hair; antennomere 5–9 sublinearly truncate with several long grooves at each innerside; aedeagus with particular shape, apicale extremely slender (figs 58 and 61–63, in Masumoto and Nishikawa 1986). China (Zhejiang, Fujian, Taiwan)	***Uloma fukiensis* Kaszab, 1954**
–	Male mentum with a pair of semi-circular hairy patches on external sides; antennomere 5–10 sublinearly truncate with one long groove at each inner side (Figs 2A–2C). China (Zhejiang)	***Uloma fengyangensis* sp. n.**

##### List of *Uloma* species from China

**(1) *Uloma acrodonta* sp. n.**

China: Zhejiang.

**(2) *Uloma bonzica* Marseul, 1876**

China: Zhejiang (new record). Korea (Masumoto and Nishikawa 1986; Kwon and Choi 1986; Löbl et al. 2008); Japan (Marseul 1876; Gebien 1940; Nakane 1956; Masumoto and Nishikawa 1986; Kwon and Choi 1986; Löbl et al. 2008).

**(3) *Uloma castanea* Ren & Liu, 2004**

China: Yunnan (Ren and Liu 2004; Löbl et al. 2008; Liu and Ren 2013); Guangxi (Liu and Ren 2007; Löbl et al. 2008); Henan, Anhui, Chongqing, Sichuan, Guizhou, Fujian (Liu and Ren 2013).

**(4) *Uloma compressa* Liu & Ren, 2008**

China: Yunnan (Liu and Ren 2008; Liu and Ren 2013); Hunan, Sichuan, Guizhou, Guangxi, Guangdong, Taiwan (Liu and Ren 2013).

**(5) *Uloma contortimargina* Liu & Ren, 2007**

China: Hunan, Yunnan, Guizhou (Liu and Ren 2013); Guangxi (Liu and Ren 2007; Liu and Ren 2013).

**(6) *Uloma contracta* Fairmaire, 1882**

China: Yunnan, Guangxi, Hainan (Liu and Ren 2007; Löbl et al. 2008; Liu and Ren 2013). Malaysia (Schawaller 2000); Indonesia (Fairmaire 1882; Gebien 1913; Gebien 1940; Schawaller 2000; Liu and Ren 2007; Liu and Ren 2013); Philippines (Gebien 1913; Gebien 1921); Oriental Region (Löbl et al. 2008).

**(7) *Uloma excisa excisa* Gebien, 1914**

China: Zhejiang (Ba and Ren 2009); Guangxi (Liu and Ren 2007; Löbl et al. 2008); Hainan (Löbl et al. 2008); Fujian (Kaszab 1954; Löbl et al. 2008); Taiwan (Gebien 1914; Gebien 1940; Masumoto and Nishikawa 1986; Löbl et al. 2008; Akita and Masumoto 2015); SE China (Akita and Masumoto 2015). Vietnam (Kaszab 1980; Masumoto and Nishikawa 1986; Akita and Masumoto 2015); Korea (Kim and Kim 2004); Japan (Nakane 1956; Akita and Masumoto 2015); Oriental Region (Löbl et al. 2008).

**(8) *Uloma fengyangensis* sp. n.**

China: Zhejiang.

**(9) *Uloma formosana* Kaszab, 1941**

China: Taiwan (Kaszab 1941; Masumoto and Nishikawa 1986; Löbl et al. 2008).

**(10) *Uloma fukiensis* Kaszab, 1954**

China: Zhejiang (Fang and Wu 2001; Ren and Dong 2001; Ba and Ren 2009); Fujian (Kaszab 1954; Löbl et al. 2008); Taiwan (Masumoto and Nishikawa 1986; Löbl et al. 2008).

**(11) *Uloma gongshanica* Ren & Liu, 2004**

China: Yunnan (Ren and Liu 2004; Löbl et al. 2008; Liu and Ren 2013); Hubei, Guizhou, Fujian, Taiwan (Liu and Ren 2013).

**(12) *Uloma hirticornis* Kaszab, 1980**

China: Yunnan (Kaszab 1980; Liu and Ren 2013). Vietnam (Kaszab 1980).

**(13) *Uloma integrimargina* Liu & Ren, 2007**

China: Guangxi (Liu and Ren 2007).

**(14) *Uloma intricornicula* Liu, Ren & Wang, 2007**

China: Guangxi (Liu and Ren 2007); Fujian (Liu et al. 2007).

**(15) *Uloma kondoi* Nakane, 1968**

China: Fujian (Liu et al. 2007). Japan (Nakane 1968; Masumoto and Nishikawa 1986; Liu et al. 2007; Löbl et al. 2008).

**(16) *Uloma liangi* Ren & Liu, 2004**

China: Yunnan (Ren and Liu 2004; Löbl et al. 2008; Liu and Ren 2013); Anhui, Chongqing, Sichuan, Guizhou, Fujian (Liu and Ren 2013).

**(17) *Uloma longolineata* Liu & Ren, 2007**

China: Guangxi (Liu and Ren 2007).

**(18) *Uloma meifengensis* Masumoto, 1982**

China: Taiwan (Masumoto 1982; Masumoto and Nishikawa 1986; Löbl et al. 2008).

**(19) *Uloma metogana* Ren & Yin, 2004**

China: Tibet (Ren and Yin 2004; Liu and Ren 2013); Yunnan (Liu and Ren 2013); Guangxi (Liu and Ren 2007; Liu and Ren 2013).

**(20) *Uloma minuta* Liu, Ren & Wang, 2007**

China: Henan, Anhui, Hunan, Sichuan, Yunnan, Guangdong (Liu and Ren 2013); Guangxi (Liu and Ren 2007); Fujian (Liu et al. 2007).

**(21) *Uloma miyakei* Masumoto & Nishikawa, 1986**

China: Taiwan (Masumoto and Nishikawa 1986; Löbl et al. 2008).

**(22) *Uloma mulidenta* Ren & Liu, 2004**

China: Yunnan (Ren and Liu 2004; Löbl et al. 2008; Liu and Ren 2013); Chongqing, Guizhou (Liu and Ren 2013).

**(23) *Uloma nakanei* Masumoto & Nishikawa, 1986**

China: Taiwan (Masumoto and Nishikawa 1986; Löbl et al. 2008).

**(24) *Uloma nanshanchica* Masumoto & Nishikawa, 1986**

China: Taiwan (Masumoto and Nishikawa 1986; Löbl et al. 2008).

**(25) *Uloma nomurai* Masumoto, 1982**

China: Taiwan (Masumoto 1982; Masumoto and Nishikawa 1986; Löbl et al. 2008).

**(26) *Uloma polita* (Wiedemann, 1821)**

China: Zhejiang (Fang and Wu 2001; Ren and Dong 2001); Guangxi (Liu and Ren 2007; Löbl et al. 2008); Taiwan (Miwa 1931; Gebien 1940; Masumoto and Nishikawa 1986; Schawaller 1996; Löbl et al. 2008). India (Gebien 1912; Miwa 1931; Gebien 1940; Masumoto and Nishikawa 1986; Schawaller 1996; Löbl et al. 2008); Sri Lanka (Walker 1858; Miwa 1931; Masumoto and Nishikawa 1986; Schawaller 1996); Nepal (Schawaller 1996; Löbl et al. 2008); Bhutan (Kaszab 1975; Schawaller 1996; Löbl et al. 2008); Bangladesh (Wiedemann 1821); Burma (Gebien 1912; Masumoto and Nishikawa 1986; Schawaller 1996); Thailand (Schawaller 1996); Laos (Gebien 1912); Vietnam (Gebien 1912; Kaszab 1980; Schawaller 1996); Indonesia (Miwa 1931; Gebien 1940); Japan (Masumoto and Nishikawa 1986; Schawaller 1996; Löbl et al. 2008); Madagascar (Fairmaire 1903; Gebien 1940; Masumoto and Nishikawa 1986; Schawaller 1996; Schawaller 2015); Mauritius (Gebien 1940; Masumoto and Nishikawa 1986; Schawaller 1996; Schawaller 2015); Rodriguez Islands (Schawaller 1996; Schawaller 2015); Himalayas (Schawaller 1996); Oriental Region (Löbl et al. 2008; Schawaller 2015); Afrotropical Region (Löbl et al. 2008).

**(27) *Uloma quadratithoraca* Liu & Ren, 2008**

China: Hunan (Liu and Ren 2008).

**(28) *Uloma reitteri* Kaszab, 1941**

China: Sichuan (Kaszab 1941b; Löbl et al. 2008).

**(29) *Uloma reticulata* Liu, Ren & Wang, 2007**

China: Fujian (Liu et al. 2007).

**(30) *Uloma rubripes rubripes* (Hope, 1831)**

China: Taiwan (Miwa 1931; Löbl et al. 2008). India (Gebien 1940; Schawaller 1996; Schawaller 2000; Löbl et al. 2008); Nepal (Hope 1831; Gebien 1927; Schawaller 1996; Löbl et al. 2008); Bhutan (Kaszab 1975; Schawaller 1996; Löbl et al. 2008); Thailand (Gebien 1927; Schawaller 1996); Vietnam (Fairmaire 1893; Kaszab 1980); Malaysia (Schawaller 1996; Schawaller 2000); Indonesia (Fabricius 1801; Miwa 1931; Fairmaire 1882; Gebien 1914; Gebien 1927; Schawaller 1996; Schawaller 2000); Philippines (Gebien 1921; Gebien 1940; Schawaller 2000); New Guinea (Gebien 1940; Schawaller 2000); Himalayas (Schawaller 1996; Schawaller 2000); Oriental Region (Löbl et al. 2008); Australian Region (Löbl et al. 2008).

**(31) *Uloma sauteri* Kaszab, 1941**

China: Taiwan (Kaszab 1941a; Masumoto and Nishikawa 1986; Löbl et al. 2008).

**(32) *Uloma splendida* Ren & Liu, 2004**

China: Yunnan (Ren and Liu 2004; Löbl et al. 2008; Liu and Ren 2013); Guizhou (Liu and Ren 2013).

**(33) *Uloma takagii* Masumoto & Nishikawa, 1986**

China: Taiwan (Masumoto and Nishikawa 1986; Löbl et al. 2008).

**(34) *Uloma tsugeae* Masumoto, 1982**

China: Taiwan (Masumoto 1982; Masumoto and Nishikawa 1986; Löbl et al. 2008).

**(35) *Uloma valgipes* Liu & Ren, 2013**

China: Yunnan (Liu and Ren 2013).

**(36) *Uloma versicolor* Ren & Liu, 2004**

China: Yunnan (Ren and Liu 2004; Löbl et al. 2008; Liu and Ren 2013); Guizhou (Liu and Ren 2013).

**(37) *Uloma zhengi* Liu & Ren, 2007**

China: Guangxi (Liu and Ren 2007).

## Acknowledgements

We are grateful to Dr. Kimio Masumoto (Tokyo, Japan), Dr. Shûhei Nomura of the National Museum of Nature and Science (Tokyo, Japan) and Dr. Antoine Mantilleri of Muséum National d’Histoire Naturelle (Paris, France) for the permission to examine specimens. In addition, we would like to express our gratitude to Dr. Wolfgang Schawaller (Stuttgart, Germany) for the advice about the identification of *Uloma acrodonta* sp. n. during the visit of Shanshan Liu in Staatliches Museum für Naturkunde. Thanks are due to Dr. Zhao Pan of Hebei University (Baoding, China) for valuable advice. This study was supported by the National Natural Science Foundation of China (31402003), the Science and Technology Programs for University by the Hebei Educational Committee (QN20131042) and the Key Laboratory of Zoological Systematics and Application of Hebei, China (14967611D).

## Supplementary Material

XML Treatment for Uloma fengyangensis

XML Treatment for Uloma acrodonta

XML Treatment for Uloma bonzica

## References

[B1] Akita K Masumoto K (2015) New or little-known Tenebrionid species (Coleoptera) from Japan (17) Descriptions of new taxa, proposal for new taxonomical treatments and new occurrence records. Elytra, Tokyo, New Series 5(2): 409–428.

[B2] Ba YB Ren GD (2009) Tenebrionidae. In: Wang YP (Ed.) Insects and forest health assessment of Wuyanling from Zhejiang. Science Press, Beijing, 275 pp.

[B3] Dejean PFMA (1821) Catalogue de la collection de coléoptères de M. le Baron Dejean. Crevot, Paris, 136 pp.

[B4] Fabricius JC (1801) Systema eleutheratorum secundum ordines, genera, species adiectis synonymis, locis, observationibus, descriptionibus. Tomus I. Bibliopolii Academici Novi, Kiliae, 506 pp.

[B5] Fairmaire L (1882) Coléoptères Hétéromères de Sumatra. Notes from the Leyden Museum 4: 219–265.

[B6] Fairmaire L (1893) Contributions a la faune indo-chinoise. 11^e^ Mémoire. Annales de la Société Entomologique de France 62: 19–38.

[B7] Fairmaire L (1903) Matériaux pour la faune coléoptérologique de la région malgache. 16^e^ note. Annales de la Société Entomologique de France 72: 181–259.

[B8] Fang ZG Wu H (2001) Tenebrionidae. In: Fang ZG, Wu H (Eds) A Checklist of Insects from Zhejiang. China Forestry Publishing House, Beijing, 452 pp.

[B9] Gebien H (1912) Neue Käfer aus der Familie Tenebrionidae des Museum Wiesbaden. Jahrbücher des Nassauischen Vereins für Naturkunde 65: 232–248.

[B10] Gebien H (1913) Die Tenebrioniden der Philippinen. The Philippine Journal of Science 8: 373–433.

[B11] Gebien H (1914) H. Sauter’s Formosa-Ausbeute. Tenebrionidae (Coleopt.). Archiv für Naturgeschichte A 79(9): 1–60.

[B12] Gebien H (1921) Philippine Tenebrionidae II. The Philippine Journal of Science 19: 439–515.

[B13] Gebien H (1927) Fauna Sumatrensis (Beitrag Nr. 31) Tenebrionidae (CoL). Supplementa Entomologica 15: 22–58.

[B14] Gebien H (1940) Katalog der Tenebrioniden, Teil II. [Part.] Mitteilungen der Münchener Entomologischen Gesellschaft 30: 755–786. [562–593]

[B15] Hope FW (1831) Synopsis of the new species of Nepaul insects in the collection of Major General Hardwicke. In: Gray JE (Ed.) The Zoological Miscellany (Vol. 1). Treuttel, Wurtz and Co., London, 40 pp.

[B16] Kaszab Z (1941a) Tenebrioniden aus Formosa (Col.). Stettiner Entomologisehe Zeitung 102: 51–72.

[B17] Kaszab Z (1941b) Neue orientalische Tenebrioniden (Coleoptera). Arbeiten über Morphologische und Taxonomische Entomologie aus Berlin Dahlem 8: 118–127.

[B18] Kaszab Z (1954) Über die von Herm J. Klapperich in der chinesischen Provinz Fukien gesammelten Tenebrioniden (Coleoptera). Annales Historico-Naturales Musei Nationalis Hungarici (S. N. ) 5: 248–264.

[B19] Kaszab Z (1975) Ergebnisse der Bhutan-Expedition 1972 des Naturhistorischen Museums in Basel. Coleoptera: Fam. Tenebrionidae. Entomologia Basiliensia 1: 313–333.

[B20] Kaszab Z (1980) Angaben zur Kenntnis der Tenebrioniden Nordvietnams (Coleoptera). Annales Historico- Naturales Musei Nationalis Hungarici 72: 169–221.

[B21] Kim JI Kim SY (2004) Taxonomic review of the tribe Ulomini (Coleoptera, Tenebrionidae) in Korea. Entomological Research 34(4): 277–281. doi: 10.1111/j.1748-5967.2004.tb00123.x

[B22] Kwon YJ Choi YS (1986) Check list of family Tenebrionidae from Korea. Insecta Koreana 6(1): 105–113.

[B23] Linnaeus C (1758) Systema naturae per regna tria naturae, secundum classes, ordines, genera, species, cum characteribus, differentiis, synonymis, locis (Tomus 1). Ed. Decima, Reformata. Laurentii Salvii, Holmiae, 823 pp.

[B24] Liu SS Ren GD (2007) Taxonomic study of the genus *Uloma* Dejean from Guangxi in China (Coleoptera, Tenebrionidae, Ulomini). Acta Zootaxonomica Sinica 32(3): 530–538.

[B25] Liu SS Ren GD (2008) Two new species of the genus *Uloma* Dejean, 1821 from China (Coleoptera, Tenebrionidae, Ulomini). Acta Zootaxonomica Sinica 33(3): 498–501.

[B26] Liu SS Ren GD (2013) Taxonomy of the genus *Uloma* Dejean (Coleoptera, Tenebrionidae, Ulomini) from Yunnan, China. Acta Zootaxonomica Sinica 38(3): 559–565.

[B27] Liu SS Ren GD Wang JS (2007) Three new species of the genus *Uloma* Dejean, 1821 from Wuyi Mountain in China, with a new record (Coleoptera, Tenebrionidae, Ulomini). Acta Zootaxonomica Sinica 32(1): 70–75.

[B28] Löbl I Merkl O Ando K Bouchard P Lillig M Masumoto K Schawaller W (2008) Tenebrionidae. In: Löbl I Smetana A (Eds) Catalogue of Palaearctic Coleoptera, Volume 5. Tenebrionoidea. Apollo Books, Stenstrup, 105–352.

[B29] Marseul SAde (1876) Coléoptères du Japon recueillis par M. Georges Lewis. Énumération des Hétéromères avec la description des espèces nouvelles. Annales de la Société Entomologique de France (5) 6: 93–142.

[B30] Masumoto K (1982) Tenebrionidae of Formosa (4). Elytra 10: 17–32.

[B31] Masumoto K Nishikawa N (1986) A revisional study of the species of the genus *Uloma* from Japan, Korea and Taiwan (Tenebrionidae, Coleoptera). Insecta Matsumurana (NS) 35: 17–43.

[B32] Matthews EG Bouchard P (2008) Tenebrionid beetles of Australia: description of tribes, keys to genera, catalogue of species. Australian Biological Resources Study, Canberra, 398 pp.

[B33] Miwa Y (1931) A systematic catalogue of Formosan Coleoptera. Reports of the Department of Agriculture of the Government Research Institute, Taihoku 55: 1–359.

[B34] Nakane T (1956) New or little-known Coleoptera from Japan and its adjacent Regions, XIII. The Scientific Reports of the Saikyo University (A) 2(3): 159–174.

[B35] Nakane T (1968) New or little known Coleoptera from Japan and its adjacent regions, XXVII. Fragmenta Coleopterologica 19–21: 76–85.

[B36] Ren GD Dong SH (2001) Tenebrionidae. In: Wu H, Pan CW (Eds) Insects of Tianmushan national nature reserve. Science Press, Beijing, 764 pp.

[B37] Ren GD Liu SS (2004) Six new species of the genus *Uloma* from Gaoligong Mountain in China (Coleoptera, Tenebrionidae). Acta Zootaxonomica Sinica 29(2): 296–304.

[B38] Ren GD Yin H (2004) Tenebrionidae. In: Yang XK (Ed.) Insects of the Great Yarlung Zangbo Canyon of Xizang. China Science and Technology Press, Beijing, 339 pp.

[B39] Schawaller W (1996) The genus *Uloma* Dejean (Coleoptera: Tenebrionidae) in the Himalayas. Acta Zoologica Academiae Scientiarum Hungaricae 42(2): 111–125.

[B40] Schawaller W (2000) The genus *Uloma* Dejean (Coleoptera: Tenebrionidae) in Borneo and Sumatra. Stuttgarter Beitraege zur Naturkunde Serie A (Biologie) 605: 1–23.

[B41] Schawaller W (2015) The genus *Uloma* Dejean (Coleoptera: Tenebrionidae: Tenebrioninae) in Africa south of the Sahara. Stuttgarter Beiträge zur Naturkunde A, Neue Serie 8: 195–206.

[B42] Walker F (1858) Characters of some apparently undescribed Ceylon insects. The Annals and Magazine of Natural History (3) 2: 202–209, 280–286.

[B43] Wiedemann CRW (1821) Neue exotische Käfer. Magazin der Entomologie 4: 107–183.

